# Indications and Complications of Hepatic Resection Patients at Sher-I-Kashmir Institute of Medical Sciences: An Observational Study

**DOI:** 10.7759/cureus.19713

**Published:** 2021-11-18

**Authors:** Irfan Kar, Kaif Qayum, Junaid Sofi

**Affiliations:** 1 General Surgery, Wye Valley NHS Foundation Trust, Hereford, GBR; 2 General Surgery, Sher-I-Kashmir Institute of Medical Sciences, Srinagar, IND

**Keywords:** observational, complications, indications, liver resection, hepatic resection

## Abstract

Aim: This study aimed to determine the indications and demographic profile of hepatic resection at Sher-I-Kashmir Institute of Medical Sciences (SKIMS), the performed types of hepatic resection, as well as assess the details of the operation and perioperative complications of hepatic resection.

Methods: This is a prospective, retrospective observational study. The retrospective study period was from January 2005 to August 2015 and the prospective study period was from 2015 till 2017. Prospective patients were clinically evaluated by medical history and clinical examination and also underwent various investigations. The patients were scored on Child-Pugh and American Society of Anesthesiology (ASA) scores for risk stratification and prepared for surgery, which included segmentectomy to major liver resection. The retrospective data were obtained from the Medical Records Department (MRD). Statistical analysis was done on SPSS software 25.0 version (Armonk, NY: IBM Corp.).

Results: This study included 122 patients with a male to female ratio of 1:1.59. The patients' age was between 1 and 73 years. The patients' most common complaint was right upper quadrant abdominal pain. The main established clinical diagnosis was oriental cholangiohepatitis (OCH) (36.9%) followed by carcinoma of gallbladder (CaGB) which accounted for 37 cases (30.4%). Liver metastases including solitary masses and multiple lesions were 10 cases (8.2%). Fifty-five patients underwent left lateral segmentectomy (45.1%) and mostly for OCH. Standard wedge resection was done in 30.7% of cases and for all cases of CaGB. The mean blood loss was 146.5 ml. A total of 37 patients had complications. Wound infection was the most common complication, occurring in 10 patients (8.2%).

Conclusion: Patients with hepatobiliary pathology, necessitating liver resection are now routinely admitted to the Department of Surgical Gastroenterology in SKIMS, Srinagar. Patients are carefully evaluated and operated with a confirmed definitive diagnosis. The overall surgical outcome does not differ from India's best centers.

## Introduction

Hepatic (liver) resection is the main cornerstone for primary and secondary liver tumors treatments showing compelling long-term oncological outcomes compared with other interventional or medical therapy in several hepatobiliary and oncological diseases [1-4]. In order to plan for liver resection, the considerations include the lesion's nature and its location within the liver, the patient's anatomy, and the quality and volume of the hepatic tissue remaining following the resection, ensuring an adequate future hepatic remnant [5-7].

Traditionally, four hepatic resections were commonly performed which include the formal left or right hepatic lobectomy, lateral segmentectomy, or trisegmentectomy (extended right hepatic lobectomy). However, due to the improvements of the functional anatomy's knowledge, liver resections have evolved [8].

Major hepatic resections are needed for both malignant conditions such as hepatocellular carcinoma (HCC), cholangiocarcinoma, and other rare types of malignant tumors, and also benign primary liver tumors including giant hemangiomas and adenomas to achieve ideal therapeutic outcomes [9]. Perioperative mortality after hepatic resection is 1-3% at high volume centers [10].

Patients having no liver cirrhosis can tolerate up to 75% of liver volume resection or up to six segments. On the other hand, patients with Child-Pugh B or C livers have high rates of complications even when undergoing minor hepatic resections [11]. The surgical morbidity of hepatic resection differs in different studies because of the differences in the categorization of complications, with estimation between 4.1% and 47.7% [12]. Common complications following hepatic resection are venous catheter-related infection, pleural effusion, incisional infection, pulmonary atelectasis or infection, ascites, subphrenic infection, urinary tract infection, intraperitoneal hemorrhage, gastrointestinal (GIT) bleeding, biliary tract hemorrhage, coagulation disorders, bile leakage, and liver failure [13]. Liver failure is the most serious complication following hepatic resection and may be life-threatening [14,15].

This study aimed to determine the indications and demographic profile of hepatic resection at our Sher-i-Kashmir Institute of Medical Sciences (SKIMS), the types of hepatic resection performed during the study period, and assesses the details of the operation and perioperative complications of hepatic resection.

## Materials and methods

Study design, site, and period

This was a prospective, retrospective observational study. This study was conducted in the Department of Surgical Gastroenterology, General and Minimal Invasive Surgery, Pediatric Surgery, and Surgical Oncology, SKIMS, Soura, Srinagar, the tertiary care hospital in Jammu and Kashmir. The retrospective study period was from January 2005 to August 2015 and the prospective study was from 2015 till 2017, a two-year period.

Methodology

Prospectively, each patient, whether admitted electively or in emergency, was clinically evaluated by medical history and clinical examination. Then, there were investigations of the complete blood count, coagulogram, biochemical workup, and study of imaging (USG, CT/magnetic resonance cholangiopancreatography {MRCP}). The preliminary diagnosis was established based on the investigations, and the surgical resection was planned accordingly. The patients were optimized preoperatively for any need of blood transfusion or correction of coagulopathy. The patients were scored on Child-Pugh and American Society of Anesthesiology (ASA) scores for risk stratification and prepared for surgery, which comprised of segmentectomy to major liver resection.

Also, retrospectively, files were obtained from the medical records department (MRD) and studied with collecting all relevant data. The postoperative morbidity and mortality were recorded till discharge from the hospital and in the follow-up period up to 30 days after discharge from the hospital.

Exclusion criteria and statistical analysis

We excluded patients with severe medical morbidity as such not fit for surgery and those with Child C criteria. Data analysis was performed using SPSS software. The data are presented as mean±SD, in absolute numbers, and as percentages.

## Results

Baseline characters

In this study, 122 patients were included. The total males were 38.5% (47 cases), with a male to female ratio of 1:1.59. The patients' age was between 1 and 73 years (mean±SD=41.1±15.97); 54.7% were in the age group of 15-50 years and 38.5% cases were in the elderly age group (>50 years). The patients' most common complaint was abdominal pain, more in the right upper quadrant, 59.8%. Other signs included jaundice (11.5%) and fever (9.9%), and symptoms included nausea (9.9%) and weight loss (5.6%). Four (3.3%) patients were asymptomatic (Table 1).

**Table 1:** Baseline characteristics of the study patients

Characteristic	Number (%)
Age<15 years	5 (4.1%)
Age 15-50 years	70 (57.4%)
Age≥50 years	47 (38.5)
Right upper quadrant pain	73 (59.8%)
Jaundice	14 (11.5%)
Fever	12 (9.9%)
Nausea	12 (9.9%)
Weight loss	7 (5.6%)
Asymptomatic	4 (3.3%)

Preoperative investigations for assessment and planning

Hemoglobin, leucocyte count, bilirubin, serum albumin, aspartate aminotransferase (AST), alanine transaminase (ALT), alkaline phosphatase (ALP), and coagulogram were taken as laboratory parameters to assess the patient’s general condition and state of liver function before surgery. Child score was the main criterion of the patients considered for liver resection.

The mean hemoglobin and platelet count were 11.57 g/dl and 1.04 lakhs per microliter, respectively. The highest observed level of serum bilirubin was 32.9 mg/dl and the lowest was 0.1 mg/dl. The liver enzymes were calculated and mean AST and ALT were 37 IU/l and 52 IU/l, respectively. The mean ALP observed was 197 IU/l. The mean albumin was 3.7 mg/dl. The mean coagulogram including prothrombin time (PT), activated partial thromboplastin time (aPTT), and international normalized ratio (INR) were within the normal limits (Table 2). Only three patients were Child B and the rest were Child A.

**Table 2:** Lab parameters of study patients HB: hemoglobin; PT: prothrombin time; aPTT: activated partial thromboplastin time; INR: international normalized ratio; AST: aspartate aminotransferase; ALT: alanine transaminase; ALP: alkaline phosphatase

Lab parameter	Mean (SD)	Minimum-maximum
HB	11.57 (2.07)	6.4-17.3
PT	14.21 (2.18)	11-26
aPTT	30.54	9.17-3 90
INR	1.27 (0.28)	0.89-2.3
Bilirubin	2.15 (4.37)	0.1-32.9
Albumin	3.7 (1.3)	2.4-4.6
AST	37 (3.2)	23-98
ALT	56 (4.1)	35-156
ALP	197 (1.9)	147-298
Platelets	104 (2.4)	68-279

In all cases, initial abdominal ultrasonography was done for a preliminary diagnosis; also, contrast-enhanced computed tomography (CECT) abdomen was done with nearly consistent findings in the majority of cases with intraoperative confirmation. Magnetic resonance cholangiopancreatography (MRCP) was done in some cases to establish the diagnosis (Table 3).

**Table 3:** Imaging modalities for preoperative assessment CECT: contrast-enhanced computed tomography; MRCP: magnetic resonance cholangiopancreatography

Imaging	Number of patients	Diagnostic accuracy after intraoperative findings and histopathology	Sensitivity as per the different studies
USG abdomen	122	73%	85% to 88%
CECT abdomen	121	86%	82.8% to 91.4%
MRCP	73	99%	98%

Indications for resection

The main established clinical diagnosis was oriental cholangiohepatitis (OCH) 36.9%, followed by carcinoma of gallbladder (CaGB), accounting for 37 cases (30.4%). Liver metastases including solitary masses and multiple lesions were 10 cases in the series (8.2%). Trauma, hydatid cyst liver, and hepatic hemangiomas had equal proportion with a total of 18 cases (4.9% each). Other diagnoses included patients with bile duct injury, liver abscess, focal nodular sclerosis, hepatocellular carcinoma (HCC), cholangiocarcinoma, and hepatoblastoma, altogether accounting for 12 cases (9.8%), around one or two in each (Table 4).

**Table 4:** Diagnosis of study patients LM: liver metastases; HH: hepatic hemangiomas

Diagnosis	Number (%)
Oriental cholangiohepatitis	45 (36.9)
Carcinoma of gallbladder	37 (30.3)
LM	10 (8.2)
HH	6 (4.9)
Trauma	6 (4.9)
Hydatid	6 (4.9)
Hepatocellular carcinoma	3 (2.5)
Other diagnoses	9 (7.3)

Co-morbidities

Forty-seven patients (38.6%) had medical morbidities, and hypertension was the most common of them (27.9%), followed by diabetes mellitus (8.2%) and COPD (2.5%). Chronic liver disease was not common in our study with only three patients having it.

Liver resection type

Fifty-five (45.1%) patients underwent left lateral segmentectomy (LLS) and mostly for OCH. Standard wedge resection was done in 30.7% of cases and for all cases of CaGB. Left hepatectomy (LH) and right hepatectomy were done in 10.7% and 8.2% cases, respectively, for different pathologies involving either lobe extensively. Liver metastasectomy was performed in 5.7% of cases. It was the non-anatomical resection of the liver involving more than one segment. It was done as an isolated procedure and in a few cases in conjunction with the resection of the primary lesion in the gastrointestinal tract, and in one case as part of left hepatectomy. Right trisegmentectomy was done in one pediatric patient who was diagnosed with a case of hepatoblastoma.

Operation time and blood loss

The operation time varied depending on the indication and the procedure planned. The average procedure time was 2.6 hours (156 minutes), ranging from one to five hours. The mean blood loss was 146.5 ml, where 39.3% cases had less than 100 ml blood loss and 23% cases had more than 200 ml blood loss, with the highest blood loss of 600 ml.

Complications

A total of 37 patients had complications. Wound infection was the most common complication, occurring in 10 patients (8.2%), followed by fever (7.4%). Wound infection was seen more in patients who had bile contamination and associated diabetes mellitus, anemia, or hyponatremia. Bile leak was seen in 4.9% of cases. In this study, bile leak was more common in major resections and trauma cases. Lung complications were seen in 3.3% of cases, occurring more in the elderly and patients with underlying COPD. They included basal atelectasis, pleural infection, and pneumonia. Intraabdominal hemorrhage, ascites, and melena were seen in a few cases (1.6% each) (Table 5).

**Table 5:** Postoperative complications in study patients

Complications	Frequency	Morbidity (%)	Mortality
Wound infection	10	8.2	Nil
Fever	9	7.4	Nil
Bile leak	6	4.9	Nil
Pulmonary complications (lung complications)	5	3.3	Nil
Hemorrhage	2	1.6	Nil
Melena	2	1.6	Nil
Ascites	2	1.6	Nil
Biliary fistula	1	0.81	Nil

Length of stay

The mean hospital stay was 7.5 days with standard deviation of 4.49. The minimum stay was of three days and the maximum was 35 days. There were 48.4% of patients who stayed seven or more days, while those who stayed less than seven days in the hospital were 51.6%.

## Discussion

This study showed that the main indication for hepatic resection in SKIMS was OCH (36.9%) followed by CaGB, which accounted for 37 cases (30.4%). The majority of patients were from the North Kashmir district of Baramulla. The main resection type used was the LLS (45.1%), which was mostly performed for OCH. Thirty-seven out of 122 included patients suffered from complications. Wound infection was the commonest complication (10 patients; 8.2%), followed by fever (7.4%). The mean blood loss was 146.5 ml and the mean hospital stay was 7.5 days.

Hepatic resection needs a comprehensive understanding of the anatomy of the liver [8]. A crucial decision in any liver resection is the choice of the amount of parenchyma to be removed. Anatomic resections usually involve two or more liver segments, while non-anatomic resection involves resection of the metastasis with a margin of uninvolved tissue (segmentectomy). Even though the preoperative treatment permits more patients to be eligible for resection, it can compromise liver function and increase the risk for liver failure following the operation [16]. Therefore, choosing whether to perform a non-anatomic or wedge resection should consider several factors including the preoperative chemotherapy, pre-existing liver disease, tumor burden, and recurrence risk, and whether or not the outcome will be influenced by the extent of resection [17].

Liver resections became more prevalent after the development in general anesthesia and antibiotics. The first successful liver resection is attributed to Dr. Langenbuch in 1888, although the patient was re-operated for bleeding [18]. Perioperative outcomes for liver resection had improvements because of better surgical techniques that take advantage of the segmental anatomy of the liver, enhanced techniques for bleeding control, and improved intensive care. Hepatic resection that is performed in high-volume centers by very well-trained hepatobiliary surgeons is associated with more favorable outcomes [5-7].

The demographic differences were observed in this study. As far as the rural and urban areas are concerned, the residential area was also considered as an explanatory variable of the study. Most of the cases are reported to this institute as this is the only tertiary care hospital in the state, except a few who go outside the state for treatment. Our findings showed higher incidence rates in urban and suburban areas in comparison with rural areas. This may be mainly because the majority of our population is in these areas and small townships. Migration of the population, mainly the working class, to urban areas, may be a reason for the increasing trend of incidence in the urban population. Our incidence data provide information about the residence of the patients at the time of diagnosis only, and not about the previous residence. The large number of patients coming from certain districts can be mainly explained by the large population of these areas, increased awareness about health care, and easy access to the health care system. Many patients who belong, at present, to the recently formed districts were kept in the parent districts (as in 2005) from which these were created in order to avoid any confusing data in the study.

Blood loss is an important variable that affects the postoperative outcome following liver resection [19]. For a safely performed liver resection, it is necessary to be familiar with various available hepatic vascular occlusion techniques and to minimize blood loss and blood transfusions requirements.

The role of laparoscopy in surgery is a growing field. Currently, it is now utilized in liver resections in institutions with experiences in minimally invasive surgical techniques. There are various approaches such as total laparoscopic, hand-assisted laparoscopic, and robot-assisted liver resection [20]. The majority of them have been done by total laparoscopic method followed by hand-assisted laparoscopic method. The most common liver resections performed laparoscopically are wedge resections, followed by left lateral segmentectomy [21,22]. Rocca et al. recently concluded that robot-assisted resection surgery for colorectal cancer liver metastases could be considered as a technical improvement choice for a minimally invasive approach even for simultaneous operations and challenging cases [23].

OCH, also known as recurrent pyogenic cholangitis, is an endemic disease in Southeast Asia and is now seen globally because of increased population mobility and international travel [24]. It is characterized by the presence of multiple intraductal calculi leading to biliary tree dilatation and stricture [25]. It was the main established clinical diagnosis in this study, followed by CaGB. Eilard et al. reviewed the surgical treatment for CaGB, comparing radical resection, including liver and lymph node resection, with cholecystectomy alone. The radical resection showed significantly better survival in patients with stages T1b and above [26].

Even though this study had very few cases of HCC, it is the most common primary liver cancer. Hepatic resection has the highest local controllability resulting in long-term survival for HCC [27]. A recent study concluded that surgical management of advanced HCCs with macroscopic portal vein invasion is feasible, and associated with comparable disease-free survival; however, it has poorer overall survival, compared to patients without portal vein invasion [28].

Hepatic resection for colorectal metastases is affected by recurrence rate. In patients who underwent aggressive surgery for colorectal metastases, very early recurrence occurred in around 12% of them. Very early recurrence is associated with very bad prognosis, more likely to that of un-resected patients, and a low chance of effective repeated treatment [29].

A total of six cases (4.9%) in this study were diagnosed with hydatid liver disease. Hassan et al. conducted a study at SKIMS, studying hydatid liver disease and disseminated hydatidosis [30]. They reported a series of cases in the tertiary care hospital of SKIMS and presented a brief overview of the disease as well as the radiographs of patients. The limitations of this study include the small sample size and the retrospective design which puts the data at risk of bias. The limitations include the small sample size, and the retrospective included design which puts the data at risk of bias.

## Conclusions

Patients with hepatobiliary pathology, warranting liver resection are now routinely admitted to the Department of Surgical Gastroenterology in SKIMS. Patients are thoroughly evaluated and operated with a confirmed definitive diagnosis. The overall surgical outcome is similar to the best centers in India. Over the years and with increasing experience, these surgical procedures are now done more frequently than before. The improvements in imaging modalities, diagnostics, advanced equipment, and better postoperative care in surgical ICU and in wards can assist in enhanced patient care in this institute, SKIMS.
